# Prognostic Significance of Circulating and Endothelial Progenitor Cell Markers in Type 2 Diabetic Foot

**DOI:** 10.1155/2014/589412

**Published:** 2014-02-03

**Authors:** Maria Sambataro, Elena Seganfreddo, Fabio Canal, Anna Furlan, Laura del Pup, Monia Niero, Agostino Paccagnella, Filippo Gherlinzoni, Angelo Paolo dei Tos

**Affiliations:** ^1^Metabolism Disease and Clinical Nutrition Unit, Santa Maria di Ca' Foncello Hospital, Piazza Ospedale 1, 31100 Treviso, Italy; ^2^Department of Pathology, Santa Maria di Ca' Foncello Hospital, Treviso, Italy; ^3^Hematology Unit, Santa Maria di Ca' Foncello Hospital, Treviso, Italy; ^4^Immunohematology and Transfusion Service, Santa Maria di Ca' Foncello Hospital, Treviso, Italy

## Abstract

*Objective*. We studied circulating precursor cells (CPC) in type 2 diabetes mellitus (T2DM) with neuropathic foot lesions with or without critical limb ischemia and relationships between endothelial precursor cells (EPC) and peripheral neuropathy. *Methods and Subjects*. We measured peripheral blood CD34, CD133, and CD45 markers for CPC and KDR, CD31 markers for EPC by citofluorimetry and systemic neural nociceptor CGRP (calcitonin gene related protein) by ELISA in 8 healthy controls (C) and 62 T2DM patients: 14 with neuropathy (N), 20 with neuropathic foot lesions (N1), and 28 with neuroischemic recent revascularized (N2) foot lesions. Timing of lesions was: acute (until 6 weeks), healed, and not healed. *Results*. CD34+ and CD133+ were reduced in N, N1, and N2 versus C, and CD34+ were lower in N2 versus N1 (*P* = 0.03). In N2 CD34+KDR+ remain elevated in healed versus chronic lesions and, in N1 CD133+31+ were elevated in acute lesions. CGRP was reduced in N2 and N1 versus C (*P* < 0.04 versus C 26 ± 2 pg/mL). CD34+KDR+ correlated in N2 with oximetry and negatively in N1 with CGRP. *Conclusions*. CD34+ CPC are reduced in diabetes with advanced complications and diabetic foot. CD34+KDR+ and CD31+133+ EPC differentiation could have a prognostic and therapeutic significance in the healing process of neuropathic and neuroischemic lesions.

## 1. Introduction

Diabetic foot is, as WHO definition, a group of syndromes in which tissue breakdown occurs because of neuropathy, ischaemia, and/or infection [1]. Foot ulcer precedes 80% of various levels limb amputations and, even if with evident variability, diabetic foot is the leading cause of nontraumatic limb amputations [1, 2]. The reason of lower limb involvement and the poor prognosis in not well understood.

Diabetic macroangiopathy (DM) has some peculiar aspects: frequent coexistent coronary disease [2], longitudinal tunica media calcification, frequent endovascular occlusions below the knee, and collateral vase rarefaction [3, 4]. The consequences are chronic limb ischemia (or CLI, also described by a tissue oxygen delivery below 30 mm Hg) and a compromission of the reparative processes [5]. A controversial issue of limb salvage by endovascular angioplasty is wound healing irrespective of arterial patency and frequent vascular reocclusion [4].

Recently, it has been demonstrated that DM is characterized by a strong reduction of circulating precursor cells (CPC) CD34+ and CD133+ which have the ability to differentiate into mature endothelium and take part of vascular homeostasis and neoangiogenesis. If CPCs share markers of hemangioblastic (CD34 and CD133) and endothelial (KDR, CD31) lineages, we identify an endothelial precursor cell (EPC) [6]. KDR is a tyrosine kinase receptor of vascular endothelial growth factor 2 (VEGF2), a mediator of vascular adhesion, proliferation, and hypotensive effect in mature endothelial cells as demonstrated in animal studies [7]. CD31, also known as platelet endothelial cell adhesion molecule-1 (PECAM-1), is a 140 kDa type I transmembrane glycoprotein that is expressed at high levels on early and mature endothelial cells as in capillary microcirculation, but also in human bone marrow stem cells [8]. Recently, nonfavourable EPC modified cells of bone marrow origin were described in diabetes [9]. These authors observed that CD34+ EPC intima calcifying cells of carotid plaque are not representative of media calcifying mesenchymal cells in diabetic distal arteries [9].

Furthermore, several studies demonstrated coexistence of peripheral nervous system damage in patients with diabetes and CLI [10]. A novel aspect of diabetic neuropathy is its involvement in bone marrow function in terms of EPC impaired release, the so-called bone marrow “mobilopathy” [11, 12]. According to this hypothesis, several neuronal nociceptors, as CGRP, are recently demonstrated to be involved in human cardiovascular biology and HUVEC endothelial cells in vitro migration [13]. This 37-amino acid neuropeptide seems also to have a role in human bone marrow (BM) EPC mobilization in patients with chronic myocardial ischemia; in particular BM CD34+ exhibited various neuropeptide receptors (RAMP-1+ for CGRP) that could contribute to migration and homing of CD34+45− to ischemic cardiac regions [14].

Our aim was to evaluate EPC patterns and CGRP peripheral expression in a court of diabetic neuropathic patients with ischemic and nonischemic foot lesions identifying their possible prognostic value for clinical practice.

## 2. Materials and Methods

### 2.1. Subjects

The study protocol was approved by Local Ethics Committee of Treviso province and authorized by Local Health Department. All subjects gave written informed consent after the nature of the procedure was explained. All the procedures were performed in accordance with the Declaration of Helsinki. A total of 62 type 2 diabetic subjects (T2DM) were consecutively recruited from the outpatient Metabolic Unit of Santa Maria of Ca' Foncello Hospital. Subjects were (Table 1) nonmacrovascular (average oximetry ≥ 50 mm Hg) peripheral neuropathic (diabetic neurological index (DNI) positive and neuropathy disability score (NDS) positive [15, 16]) T2DM without foot lesions (14 N); nonmacrovascular peripheral neuropathic T2DM with foot lesions (N1 = 20); macrovascular (average oximetry ≤ 30 mm Hg) neuropathic diabetic patients with ulcers/lesions/osteomyelitis of the lower limbs diagnosed by clinical criteria [17] and undergoing predominantly distal peripheral (endovascular/surgical) revascularization (N2 = 28). Eight healthy subjects were compared as controls (C).

N2 patients were treated following a well-known diagnostic and therapeutic algorithm of revascularization as published in 2005 [2].

We recruited N1 and N2 patients if lesions were acutely treated from less than 6 weeks of occurrence. Recurrence of lesions was not exclusion criteria. Patients were defined with acute lesion (AL) if the lesions appeared within 6 weeks and blood samples were collected 5 days after surgical foot or endovascular/vascular surgery treatment; with not healed chronic lesion (CL) if blood samples were collected after six weeks and the lesion was still active or finally with healed lesion (HL) if blood samples were collected after six weeks and the lesions were already resolved. So, we define acute (AL) or healed lesions (HL) in N1 patients while AL, HL, and CL lesions in N2 patients.

Active smoking, dialysis, pregnancy, heavy myocardial insufficiency (NYIA IV class), recent (until 6 months) myocardial infarction, or ictus were exclusion criteria. The minimal diabetes duration age was five years. Subjects older than 75 years were excluded. The male sex was prevalent (17 F/53 M). The cut-off value for definition of obesity (30 kg/m^2^ body mass index) was not an exclusion criteria. T2DM were slightly older than controls, and the age difference reached statistical significance (Table 1). T2DM controlled their glycaemia with diet (1600 kcal/day: 55% carbohydrate, 20% protein, and 25% fat with less than 10 percent as saturated fat), exercise, antidiabetic drugs, or/and insulin. According to self-reporting diaries, leisure exercise, energy, and nutrient intake were not different between groups.

All patients with neuroischemic lesions had prevalent under the knee distal macroangiopathy and were hospitalized at the Treviso Ca' Foncello Hospital for endovascular or (in two patients) combined endovascular plus leg arterial by-passes. The patients reevaluated for cardiac complications with ECG and symptoms registration before the entry at the study. All minor amputations or surgical debridements were performed during hospitalization or in day surgery regimen and foot lesions were treated with specified antibiotic therapy. T2DM diabetic complications are described in Table 1. Clinical nephropathy was represented by spot microalbuminuria or 24 hours macroalbuminuria or glomerular filtration rate with MDRD method < 90 mldl/BSA [18]. Among T2DM, not recent myocardial infarction, antiaggregant platelet therapy, nephropathy, and arterial hypertension were more frequent in N2 patients. Foot lesions classification by Texas University criteria [17] resulted: in N1 patients 7 BI, 3 BII, and 10 BIII; in N2 patients 6 DI, 3 DII, and 19 DIII. Diabetic retinopathy was defined with ETDRS criteria [19] and was less frequent in N patients. Dyslipidemia and followed treatment with statins, ACE inhibitors, beta blockers, and diuretics were equally distributed in T2DM affected patients.

### 2.2. Test and Assays

In all groups, we measure two vascular indexes with VASERA VS 1000 Instrument (Fukuda Denshi Japan): ankle/brachial ratio (Winsor Index WI), obtained calculating the oscillometric curve area of systolic peak pressures, and peripheral arterial *β*-stiffness (cardio/ankle vascular index: CAVI) [20].

In N1 + N2 patients, dorsal transcutaneous allux skin oximetry and carbon dioxide tension (expressed in mm Hg) were measured with 4 chambers oximetry/laser-doppler PERIMED V 5400 instrument.

For clinic data, blood sample was measured only in the fasting sample, as well as lipids. Blood glucose was determined by glucose oxidase method (Beckman, Fullerton, CA). Glycated hemoglobin (HbA1c; upper normal range 5.8% or 40 mmol/mol with IFCC units) was determined by on-line high-pressure liquid chromatography (HPLC; C-R4A Bio-Rad, Milan, Italy) from capillary blood [21]. Serum triglycerides (TG), total cholesterol HDL-cholesterol, and LDL-cholesterol were measured with enzymatic method [22]. Microalbuminuria was calculated with nephelometric method as albumin/creatinine ratio [23].

### 2.3. Measurement of Calcitonin Gene-Related Peptide

Peripheral blood of patients was collected in sterile EDTA vacutainer and was centrifuged at 2000 rpm for 10 min. Plasma was transferred to a sterile vial to keep it at −80°C until further use. CGRP assay was done according to the manufacturer's specifications (Human CGRP ELISA kit; USCN, Wuhan, China). Absorbance was measured at 450 nm. Resultant readings were plotted on a standard curve to determine the concentration of CGRP in each sample expressed as pg/mL. The intra-assay and interassay coefficients of variation were 7 and 10%, respectively [24].

### 2.4. Characterization of Circulating EPCs by Flow Cytometry

Peripheral blood cells were analyzed for the expression of surface antigens by direct flow cytometry as previously described [25, 26]. Before being stained with specific monoclonal antibodies, cells were treated with fetal calf serum for 10 minutes, and then the samples were washed with buffer containing phosphate-buffered saline and 0.5% bovine albumin. Then, blood cells were stained with fluorescein isothiocyanate (FITC)-conjugated anti-human CD34 monoclonal antibody (mAb) (Becton Dickinson, USA), phycoerythrin (PE)-conjugated anti-human KDR mAb (R&D Systems, USA), peridinin-chlorophyll proteins (PerCP)-conjugated anti-human CD133 mAb (Miltenyi Biotec, USA), allophycocyanin-Cy7 (APC-Cy7)-conjugated anti-human CD45 mAb (Becton Dickinson, USA), and allophycocyanin (APC)-conjugated anti-human CD31 mAb (R&D Systems, USA). Control isotypes IgG1 and IgG2a Abs were obtained from Becton Dickinson.

A total of 1.000.000 events were acquired for each analysis with the instrument FACS Canto (Becton & Dickinson) and the level of progenitor cells was expressed as number of positive events per 1.000.000 total events. Data were processed with Diva Software (Becton Dickinson) following this gating strategy: in the SSC versus FSC morphological plot, we gated mononuclear cells and then examined this population for the expression of the relevant surface antigens. We first identified CD34+ cells, which were examined for the dual expression CD133 and KDR.

We analyze CD34 with CD45 to show that most (>90%) CD34+ cells express CD45 hemangioblastic marker at low intensity (CD45dim region). In the CD34+CD133+ cell gate, we also analyzed the expression of KDR to quantify triple positive cells. A unique definition of EPCs is lacking [25]; in this study we defined CD34+, CD133+, and CD34+CD45− cells as generic circulating progenitor cells (CPCs) and CD34+KDR+, CD133+KDR+, CD34+31+, and CD133+31+ cells as EPCs.

### 2.5. Statistical Analysis

Data are presented as mean value ± standard error of the mean (SE). Statistically significant differences between groups were determined using one-way analysis of variance (ANOVA) followed by Student's *t*-test after checking for normalization. Linear regression analysis was used to describe the correlation between 2 variables. Correlation coefficients were calculated as *R* ^2^ by *Z* test. *P* < 0.05 was considered statistically significant. Differences between categorical data were assessed with Chi square test.

## 3. Results

Winsor Index (WI) was significantly reduced in N2 versus N1 patients (WI in affected limb 0.87 ± 0.05 versus 1.07 ± 0.04 median ± SE *P* < 0.006) (Table 1). CAVI index was significantly elevated in chronic versus acute and healed N2 lesions (11 ± 0.5, 9.4 ± 0.6, 9.3 ± 0.4  *P* < 0.05). Oximetry was significantly reduced in N2 versus N1 (30 ± 5 versus 51 ± 5 mm Hg *P* < 0.01) and in chronic versus healed N2 lesions (20 ± 7 versus 48 ± 6  *P* < 0.01). Carbon dioxide pressure was not statistically different in N1 versus N2 lesions (42 ± 3 and 54 ± 5 mm Hg).

**Figure 1:**
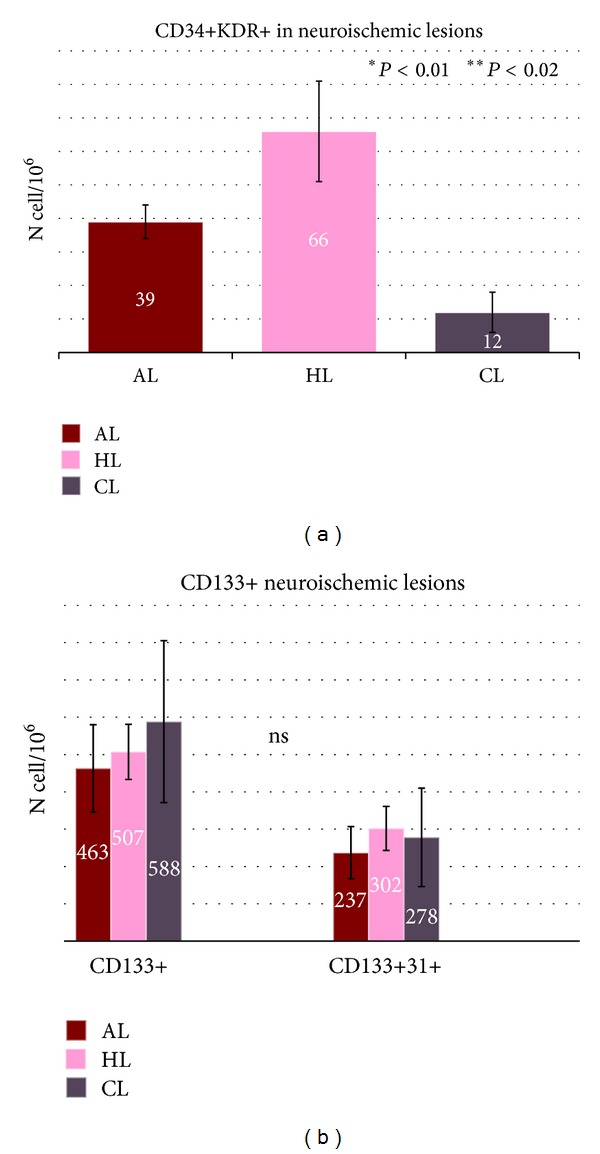
Levels of CD34+KDR+ (a) and CD133+ (b) progenitor cells in neuroischemic lesions in type 2 diabetic subjects depending of healing or not healing time course. AL acute lesions (brown), HL healed lesions (pink), and CL chronic lesions (grey). Units are N cells/10^6^  *P < 0.01 AL versus CL **P < 0.02 HL versus CL.

**Figure 2:**
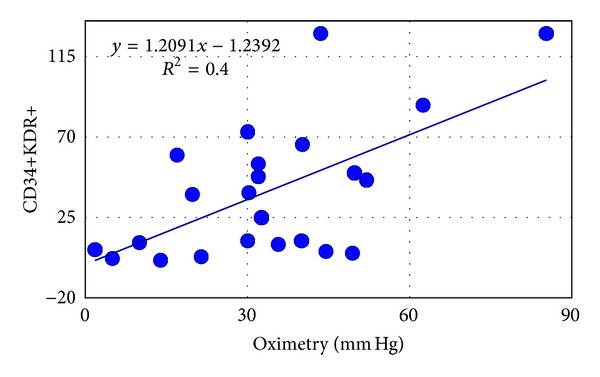
Relationships between CD34+KDR+ cells and skin foot released oxygen in type 2 diabetic neuroischemic subjects. Units for CD34+KDR+ are N cells/10^6^.

After flow cytometry analysis, CD34+ were significantly reduced in T2DM versus C (*P* < 0.03) and were significantly lower in N and N2 versus N1 (*P* < 0.03) (Table 2). In N2 patients CD34+KDR+ EPCs were positively correlated with healing of lesions (Figure 1) and postangioplastic oximetry (*P* < 0.008  *R* ^2^ = 0.41) (Figure 2). The cell populations CD34+CD45− resulted in significantly reduced N versus all other groups (C, N1, and N2, *P* < 0.04).

**Figure 3:**
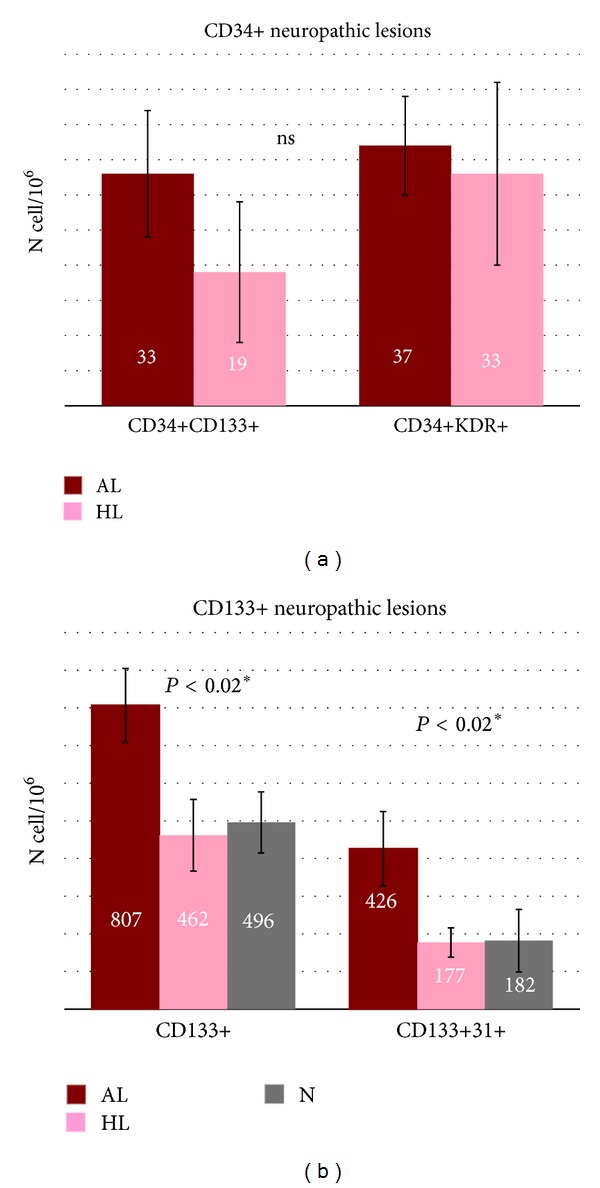
Levels of CD34+ (a) and CD133+ (b) progenitor cells in neuropathic lesions in type 2 diabetic subjects depending of healing or not healing time course. AL acute lesions (brown), HL healed lesions (pink). *P* < 0.02 in CD133+ AL in N1 patients versus HL in N1 and N patients.

CD133+, CD133+KDR+, and CD133+31+ cells were significantly reduced in all T2DM versus C (*P* < 0.0006) (Table 2). We observe an increase of CD133+ and CD133+31+ cells in acute N1 lesions without variations in CD34+KDR+ positive cells (Figure 3).

CGRP peripheral expression was significantly reduced in neuropathic patients with lesions versus C (N2 13 ± 2.2, N1 17 ± 2.2, N 18 ± 2.8, *P* < 0.004, *P* < 0.04, *P* < 0.09, resp., versus C 26 ± 2 pg/mL) and it was negatively correlated in all T2DM with CD34+CD45− (*P* < 0.01  *R* ^2^ = −0.2) and CD34+KDR+ (*P* < 0.007  *R* ^2^ = −0.2). In N1 patients CGRP better negatively correlate with CD34+CD45− (*P* < 0.0007  *R* ^2^ = −0.629) and CD34+KDR+ (*P* < 0.004  *R* ^2^ = −0.6) but did not correlated with oximetry (*P* < 0.09  *R* ^2^ = 0.1).

Fasting glycemia was significantly elevated in patients with acute neuropathic lesions (AL versus HL: 164 ± 11 versus 123 ± 16 mg/dL *P* < 0.05) and in patients with chronic neuroischemic lesions (CL versus AL: 182 ± 33 versus 119 ± 10 mg/dL *P* < 0.03) without correlation with HbA_1_c. CD133+CD31+ cells positively correlated with glycemia (*P* < 0.01  *R* ^2^ = 0.331) in neuropathic patients.

## 4. Discussion

We demonstrate in our study different roles of CPCs in neuropathic and neurovascular patients with type 2 diabetes. Neuropathic patients with foot lesions exhibit a better CD34+ homeostasis capacity to a damaged tissue than neuroischemic patients. These data could explain why in diabetes neuropathic foot lesion has a better healing prognosis with respect to ischemic foot lesions [1]. These correlations in our opinion are in accord to Asahara in vitro experiment and Li in vivo ischemic mouse model where CD34+ cells are involved in endothelial differentiation and reparative ischemic injury [27, 28]. Some researchers studied a possible role for CD34+ precursor cells in the therapy of critical lower limb ischemia CLI [29]. For example, in the Naples study, intra-arterial injected CD34+ cells of bone marrow origin were sufficient to significantly reduce time-free amputation time in a mixed diabetic and atherosclerotic ischemic group where Rutherford category 6 was exclusion criteria [30]. The number of CD34+ obtained by BM aspirate evaluated by cytofluorimetric technique (4.7 ± 3.1/10^6^ cells) seems to be lower than our CD34+ cells in neuropathic healed lesions (369 ± 34/10^6^ cells). We do not know the regenerative capacity of circulating versus reinjected CD34+ population cell. Furthermore, this result suggests an injection of more CPC that could be necessary in diabetic patients for better results.

Furthermore, in neuroischemic patients, the total CD34+ response is irrelevant also after angioplasty; only their differentiation level is a good prognosis factor for ulcer healing (Figure 1) and correlates with revascularization oximetry (Figure 2). In particular CD34+KDR+ cells increase after angioplasty and the level remains high in healed neuroischemic patients. Significative reduction of CD34+KDR+ in chronic not healed lesions probably explains frequent restenosis of distal tibial arteries, but also this hypothesis requires further investigations.

In our neuropathic patients, in normoxic conditions, CD34+KDR+ cells did not elevate after healing as acute neurovascular lesions (Figure 1). So, we hypothesized a different induction of circulating precursors in presence of neuropathy alone and we studied if neuropathy per se could have a role in vascular reactivity. First, we indirectly measured arterial stiffness with CAVI, an index which quantificates arterial wall transmission capacity of systolic peak pressure [20]. It is demonstrated that this index correlates with systemic atherosclerosis [31]. We observed a significant increment of CAVI in neuroischemic chronic nonhealed patients but we did not observed significant differences in N1 versus N2 patients.

So we decided to evaluate neural induced vascular reactivity with quantification of a typical C-fiber nociceptor, CGRP, which resulted reduced in both N1 and N2 patients. Furthermore, in N1 patients in which the CD34+ are elevated, we found a robust negative correlation between CGRP and CD34+CD45− or CD34+KDR+ and this relationship was lost in neuroischemic patients where hypoxygenation or oxidative stress or other factors probably reduced CGRP action on bone marrow CD34+ motility or VEGF-mediated angiogenesis. We did not measure neural fiber skin density as a measure of small fiber damage [32], so we do not know if CGRP secretion is functionally or irreversibly reduced in our patients. N2 revascularized patients showed a significant increment of CD34+KDR+ in healed lesions which was lost in chronic not healed lesions. So, it is possible that angioplasty therapy could partially restore per se CD34+ response in these lesions or response to CGRP, or to receptorial nociceptor efficiency in neuronal pericytes, but we do not measure this surface receptors molecules yet. The “other side of the coin” would be a reduction of CGRP action resulting in medial calcification but for this issue we need further investigations.

N1 patients had noncritical oximetry levels, did not receive endovascular/vascular surgical distal treatment, and recognize different surface CD markers for healing in CPC and EPC candidate cells. At difference with N2 group (Figure 3(a)), the N1 patients showed a significant increment of CD133+ and CD133+31+ in hyperglycemic conditions (Figure 3(b)). Generally, CD133 is considered an immature stem cell marker with multiple differentiation power irrespective of oxygenation and it could be protective against stroke [33]. CD133+ expression is lost during the differentiation to mature endothelial cells with acquisition of CD34 positivity [34, 35]. Recently, Kim and colleagues proposed CD31+ as a good marker for cardiovascular stem cell therapy due to a more conservative representation along endothelial precursors cells and hematopoietic stem cells lineage [8, 36], but, at difference with our cells, CD31+ cells were CD45+ and with probably prevalent paracrine angiogenic activity. It is possible that our clinical human conditions, with diabetic neuropathy, microfracture solicitation, hyperglycemia, hypercapnia, or CGRP reduction could induce modified CD133+ maturation. We do not know if CD133+ population in our patients is locally by foot or from bone marrow origin, or if it identifies an active granulation reparative tissue at the ulcer irrespective of pathogenesis: all these hypotheses could be an attractive issue.

## 5. Conclusions

In summary for the first time in vivo we demonstrate that diabetic neuropathic and neuroischemic foot lesions are characterized by different CPC levels and by reduced CGRP circulating levels. EPC differentiation reflects timing and healing of lesions and could have a prognostic role. The macroischemic condition and vessel patency is only one of the pathogenetic variables and this issue is crucial for new prospective views in revascularization therapy.

## Figures and Tables

**Table 1:** Clinical characteristics. 62 diabetic patients divided into 3 groups: nonmacrovascular peripheral neuropathic diabetic patients without foot lesions (N); nonmacrovascular neuropathic diabetic patients with foot lesions (N1); macrovascular neuropathic diabetic patients with ulcers/lesions/osteomyelitis of the lower limbs (N2). Eight healthy controls (C).

	C (8)	N (14)	N1 (20)	N2 (28)
Age (years)	42 ± 3	63 ± 2^∗^	58 ± 2^∗^	69 ± 2^∗^
Gender (F/M)	4/4	5/9	3/17	5/23
Smoking (y/n)	NO	NO	NO	NO
Body mass index (kg/m^2^)	28 ± 2	30 ± 1.4^∗^	31 ± 1.4^∗^	30.0 ± 1.1^∗^
Hypertension (*N*)	0	9	13	28^#^
Duration of diabetes (years)	0	9 ± 2	14 ± 3	13 ± 2
HbA1c (%; nM/M)	5.4 ± 0.5 (36 ± 1)	8.0 ± 0.5 (69 ± 6)^∗^	8.0 ± 0.3 (69 ± 6)^∗^	7.6 ± 0.3 (66 ± 6)^∗^
Fasting glucose (mg/dL)	78 ± 0.3	162 ± 14^∗^	151 ± 11^∗^	145 ± 12^∗^
Total cholesterol (mg/dL)		175 ± 14	177 ± 6.0	175 ± 9.0
LDL-cholesterol (mg/dL)		93 ± 11	94 ± 7.0	100 ± 12
HDL-cholesterol (mg/dL)		55 ± 4.0	53 ± 3.0	56 ± 5.0
Triglycerides (mg/dL)		110 ± 13	113 ± 11	133 ± 12
Winsor Index	1.06 ± 0.04^∗∗^	1.04 ± 0.4^∗∗^	1.07 ± 0.4^∗∗^	0.87 ± 0.5
Antidiabetic therapy (I/OA)		9/5	16/4	26/2
Statins (%)		50	50	60
Antiplatelet therapy (%)		43	40	100^#^
Retinopathy (%)		35	50	61^##^
Nonrecent MI/ictus (%)		7	5	42^#^
Nephropathy (%)		35	40	71^#^

*P* < 0.001 versus C; ^∗∗^ *P* = 0.006 versus N2 (ANOVA test); ^#^ *P* < 0.001  N and N1 versus N2; ^##^ *P* < 0.001  N versus N1 and N2 (Chi square test).

**Table 2:** Cytofluorimetric classes in all studied groups (mean ± SE): nonmacrovascular neuropathic diabetic patients without foot lesions (N); nonmacrovascular neuropathic diabetic patients with foot lesions (N1); macrovascular neuropathic diabetic patients with ulcers/lesions/osteomyelitis of the lower limbs (N2). Healthy controls (C).

	C (8)	N (14)	N1 (20)	N2 (28)
CD34+	515 ± 57	235 ± 18^∗#^	369 ± 34^∗°^	280 ± 24^∗^
CD133+	1970 ± 268	497 ± 81^∗∗^	686 ± 80^∗∗^	508 ± 75^∗∗^
CD34+45−	41 ± 5^∧^	21 ± 3	51 ± 9^∧^	41 ± 3^∧^
CD34+31+	273 ± 59^∧∧^	70 ± 9	175 ± 34^∧^	132 ± 19^∧^
CD133+31+	829 ± 112	248 ± 69^∗∗^	339 ± 53^∗∗^	269 ± 51^∗∗^
CD133+KDR+	122 ± 34	16 ± 5^∗∗^	26 ± 6^∗∗^	26 ± 10^∗∗^

*C  versus N, N1, N2 *P* < 0.03; ^#^N versus N1 *P* < 0.004; °N1 versus N2 *P* < 0.03; ^∗∗^C  versus N, N1, N2 *P* < 0.0006;  ^∧^N versus N1, N2, C  *P* < 0.04; ^∧∧^N2 versus C  *P* < 0.005.
